# Diabetic Ketoacidosis From Health Insurance-Requested Non-medical Switching

**DOI:** 10.7759/cureus.108059

**Published:** 2026-04-30

**Authors:** Ahmet S Can, Forrest Yeakley, Zhiwei Zhang

**Affiliations:** 1 Endocrinology, Diabetes and Metabolism, Arnot Ogden Medical Center, Elmira, USA; 2 Medicine, Arnot Ogden Medical Center, Elmira, USA; 3 Medicine, Samaritan Medical Center, Watertown, USA

**Keywords:** diabetic ketoacidosis, insulin treatment, insurance drug coverage, non-medical switching, type 1 diabetes mellitus

## Abstract

Non-medical switching is a change in treatment regimen for reasons other than efficacy, side effects, or adherence, with the purpose of decreasing healthcare costs. A 20-year-old female with type 1 diabetes mellitus presented with diabetic ketoacidosis. She was having issues with her health insurance and pharmacy in filling her prescriptions. Her health insurance changed its drug formulary after the New Year began. In response to the request of her health insurance, her existing insulin treatment was non-medically switched to a new rapid-acting insulin and a long-acting insulin. She was confused about what she should be taking. She took her newly prescribed non-medically switched long-acting insulin three times daily before meals in place of rapid-acting insulin, along with her previously prescribed long-acting insulin. Then, she could not fill her new non-medically switched prescription, because subscribers of the same health insurance were changed to the same insulin at the same time, triggering a drug shortage in our service area. She was admitted and stayed in the hospital for three days for treatment of diabetic ketoacidosis. After her discharge, her health insurance covered the original regimen that she was on before the non-medical switch. Non-medical switching defeated its purpose and increased healthcare spending in our case. Non-medical switching not only placed additional financial burden on our patient’s health, but also jeopardized patient safety by significantly contributing to an acute complication of diabetes mellitus. Further studies that compare healthcare utilization, not just drug prices, are needed to explore if non-medical switching saves money and is cost-effective. This is the first case report in the medical literature that shows that non-medical switching can cause diabetic ketoacidosis.

## Introduction

Non-medical switching is a change in treatment regimen for reasons other than efficacy, side effects, or adherence, and is often related to drug formulary changes [1]. Non-medical switching is typically enforced on healthcare providers by health insurance to contain ever-increasing healthcare spending [2]. Non-medical switching is not a global problem and occurs in certain countries where health care is not socialized. A systematic literature review criticized health insurance companies for focusing on drug prices instead of overall health resource utilization when deciding on non-medical switching for originator biologics and suggested that healthcare spending actually increases in patients who had non-medical switching [3]. Another review concluded that the majority of biosimilars are clinically equivalent to their originators, with similar efficacy and safety profiles, and endorsed their widespread use due to their lower cost [2]. There is no agreement on the value of non-medical switching in the medical care community from the provider view, although health insurance and healthcare delivery finance sectors agree on its benefits and enforce it on the patients and providers.

Diabetic ketoacidosis (DKA) is an acute, life-threatening complication of diabetes characterized by hyperglycemia, ketoacidosis, and ketonuria. We present a patient with type 1 diabetes mellitus who had been familiar with her rapid- and long-acting insulin regimen, subjected to a series of non-medical switching inflicted by her health insurance, suffered an episode of DKA that required a three-day intensive care unit stay, only to be discharged and switched back to her original insulin regimen that she was on before the non-medical switching. Non-medical switching is a widespread policy practice in the United States of America and can cause serious adverse outcomes. We try to answer the question of “Did the non-medical switching, which is set out to save on healthcare costs, really achieve its goal?”

This report was presented as a poster at ENDO 2023 in Chicago, IL, USA on June 17th, 2023.

## Case presentation

A 20-year-old female with a history of type 1 diabetes came to the emergency room with a five-day course of headache, nausea, vomiting, polyuria, and polydipsia. She stated that she ran out of insulin because there were problems between her insurance company and the pharmacy in filling her prescriptions. Both her long-acting insulin (Basaglar, Eli Lilly and Company, Indianapolis, IN, USA) and rapid-acting insulin (Admelog, sanofi-aventis U.S. LLC, Bridgewater, NJ, USA) were no longer covered. Her managed care Medicaid health plan sent a letter to our endocrinology office a few weeks before the New Year announcing that Semglee (Mylan Pharmaceuticals Inc., a Viatris Company, Morgantown, WV, USA) as long-acting and Humalog (Eli Lilly and Company, Indianapolis, IN, USA) as rapid-acting insulin, were preferred and will be covered, whereas her standing insulin prescriptions will not be covered. The insulin prescriptions were changed as requested by her insurance company, and prescriptions were transmitted to the pharmacy electronically from the electronic health record. The patient was confused about what she should be taking. This led her to think that her new long-acting insulin (Semglee) was her rapid-acting insulin due to a name that she did not recognize from her past diabetes self-management education. The patient endorsed taking long-acting insulin Semglee three times daily before meals in place of rapid-acting insulin along with the previously prescribed long-acting insulin Basaglar twice daily before breakfast and at bedtime. To make things worse, she could not refill her prescription for Semglee due to a Semglee shortage in the preceding three weeks before her admission. She did not go to the pharmacy to pick up Humalog or Admelog, although she had a prescription for both. She thought that she was meeting her rapid-acting insulin need from Semglee.

Upon presentation to the emergency room, her physical examination showed an alert and oriented patient with a blood pressure of 109/64 mmHg, pulse rate of 96 beats per minute, dry mucous membranes, and decreased skin turgor. Her serum glucose was 576 mg/dL (reference: less than 200 mg/dL). The laboratory findings are summarized in Table 1 and show mild DKA.

**Table 1:** Laboratory findings

Parameter	Result (mmol/L)	Reference range (mmol/L)
Random glucose	32	<11.1
Beta-hydroxybutyrate	6.56	<0.28
Bicarbonate	18	21-31
Anion gap	22	7-15

She was admitted to the intensive care unit, treated successfully by following standard DKA treatment protocol, and discharged three days later. After the DKA episode, she was prescribed Admelog and Basaglar, the original insulin regimen before the non-medical switch request, and now they were covered. We called her pharmacy after her DKA admission and verified the sequence of events that led to DKA. Her existing insulin regimen and that requested by her insurance are shown in Table 2.

**Table 2:** Existing insulin prescription and non-medical switching requested by the health insurance, chronologically starting six months prior to DKA, which was at the end of March 2021 DKA: diabetic ketoacidosis; Rx: prescription; Filled: insurance-requested drug dispensed by the pharmacy and used by the patient; Not Filled: not dispensed by the pharmacy or not picked up by the patient. The key transition point that predisposed to DKA is highlighted in italic.

Insulin type	Existing Rx	Insurance-requested non-medical switching Rx	Date of Rx	Outcome
Long-acting	Basaglar	None	10/19/20	Filled
Rapid-acting	Admelog	None	10/19/20	Filled
Long-acting	Basaglar	Semglee	12/30/20	Filled
Long-acting	Semglee	None	02/25/21	Filled
Rapid-acting	Admelog	Humalog	03/04/21	Not filled, not picked up
Long-acting	Semglee	None	03/04/21	Not filled, Semglee shortage
Long-acting	Semglee	Basaglar	03/04/21	Not filled, not picked up
Rapid-acting	Humalog	Lispro	03/04/21	Not filled, both not covered
Rapid-acting	Lispro	Admelog	03/04/21	Not filled, not picked up

She thought that Semglee was her rapid-acting insulin and used it as such. She did not fill her prescription for rapid-acting insulin because she erroneously thought that she was getting rapid-acting from Semglee, which is a long-acting insulin. Then her prescriptions for rapid-acting insulin were not covered, and she could not purchase Semglee from the pharmacy due to the Semglee shortage. Not asking the patient to come for a visit to explain the non-medical switch was a provider-level factor that contributed to the outcome of DKA. The key clinical error was the use of long-acting insulin in place of rapid-acting insulin, a patient-level factor. The system-level factor that triggered the adverse outcome was the non-medical switching of insulin therapy and the unavailability of the non-medically switched insulin in the pharmacy.

## Discussion

To our knowledge, this is the first case report in the medical literature that shows non-medical switching can cause DKA. Our case clearly demonstrates a temporal association between insurance-driven medication changes, patient confusion, and the development of DKA. Our patient presented with DKA, after being subjected to a series of non-medical insulin switches requested by her health insurance within a span of three months that landed her in the medical intensive care unit for three days. After her discharge from the hospital, her insurance requested her treatment to be switched back to her original insulin regimen that she was on before the non-medical switch.

Health insurance companies employ several strategies, like non-medical switching, fail-first (also known as step therapy), high out-of-pocket expenses, or complex appeal processes to combat high drug prices. Non-medical switching is typically enforced on healthcare providers by health insurance companies. It is executed by either removing the current medication from the health plan formulary or increasing the out-of-pocket costs to an unaffordable level [4]. In theory, the decision whether to switch falls on the physician, but patients cannot afford uncovered medications if the healthcare provider insists on not switching.

Non-medical switching may contribute to adverse outcomes within a multifactorial context. Provider or patient scheduling challenges, potential long geographic distances for rural patients, and the need for medical transportation for those who cannot transport themselves are some of the barriers to an additional visit to explain the new insurance-requested treatment regimen to the patient. Therefore, non-medical switching usually takes place over the electronic health record, leaving the patient out of a face-to-face conversation, predisposing confusion in the treatment regimen, as happened in our patient. Confusion about medical regimen is a treatment-related barrier that leads to increased hospitalizations [5]. As the standing and insurance-requested insulins were biosimilar, we did not ask the patient to come for a visit. An additional visit would defeat the cost-containment purpose of non-medical switching.

As many patients from the same managed care Medicaid plan change prescriptions around the same time, typically shortly after January 1st of every year, non-medical switching can trigger drug shortages, as was the case for the Semglee shortage in our service area. Our patient could not fill her prescription for Semglee, contributing to her developing DKA.

The wholesale acquisition cost of a five-pack of 3 mL (1500 Units) of Basaglar is $326.36 [6] and unbranded Semglee $147.98 [7], allowing a saving of $0.11982 (~$0.12) for one unit of insulin. Our patient was 98 kg. She is expected to consume 14,600 units of long-acting insulin per year, considering that she was on 40 units of long-acting insulin daily. By changing from Basaglar to Semglee, 1736 US Dollars would be saved in insulin expense each year. The average cost of DKA per admission is $29,981 in the US [8]. In this case, non-medical switching caused an extra expense of $28,245.

Our report shows that non-medical switching can be a cause of DKA and is a cause of confusion about the medical regimen. No savings were achieved by changing insulin for a brief time, and the patient was exposed to a potentially life-threatening DKA in the interim. We think health insurance, Centers for Medicare & Medicaid Services, in this instance, needs to reassess the unintended consequences and cost-effectiveness of the practice of non-medical switching.

Non-medical switching affects millions of patients with diabetes. Non-medical switching can pave the way for suboptimal adherence to insulin therapy due to increased emotional burden [4]. The nocebo effect is defined as negative expectations toward therapy change. Non-medical switching may trigger a negative sentiment in patients who are reluctant to switch, which can adversely impact the treatment outcomes [9]. Non-medical switching can cause a nocebo effect, resulting in poor patient adherence to therapy, with consequences of worsening glycemic control and chronic diabetic complications [9]. For example, it was reported that 53% of patients who were requested to switch from canagliflozin abandoned their therapy and did not switch [10].

A physician survey reported that utilization management procedures such as prior authorizations, step therapy, and non-medical switching were significant barriers to care and contributed to physician feelings of burnout [11]. A survey of physicians showed that 80% of endocrinologists were exposed to non-medical switching requests. The general sentiment on non-medical switching among endocrinologists and other specialists was that non-medical switching decreases medication effectiveness, increases side effects, causes increases in non-office visits, and increases phone calls with pharmacies [12]. A survey conducted on patients with diabetes showed that 20% of patients reported a moderate or high level of negative impact on their blood glucose levels, diabetes control, mental well-being, general health, and control over health after non-medical switching [13]. Eight percent of patients who switched reported recurrence of old symptoms and 14% new side effects [13].

Non-medical switching and its negative effects on clinical outcomes have also been reported in other chronic illnesses. A patient survey on asthma and chronic obstructive pulmonary disease revealed that about 60% of patients were not well controlled, and about 70% required reliever inhalers after non-medical switching [14]. A real-world study in hidradenitis suppurativa showed that drug-naïve patients who were started on biosimilar adalimumab and those who were on and switched from originator adalimumab experienced lower clinical response, higher loss of clinical response, and a shorter time to loss of response [15]. In contrast, there are studies that show no negative consequences with non-medical switching, although none of them pertain to diabetes mellitus. In a multicenter cohort study, there was no difference in remission rates, compliance, and side effects between the originator infliximab and its biosimilar in patients with inflammatory bowel disease [16]. A province-wide study from British Columbia, Canada, found no increase in health resource utilization as assessed by the cost of physician services, hospitalizations, emergency room visits, and concomitant drug use for inflammatory arthritis in patients with inflammatory arthritis who were non-medically switched from infliximab, etanercept, or adalimumab. As biosimilar prices were confidential, a comprehensive cost-effectiveness analysis could not be performed. There was no increase in the evaluated healthcare costs for patients who were switched to biosimilars, implying savings [17]. Other studies reported no difference between the originator biologics and their biosimilars in a variety of clinical disorders, including rheumatoid arthritis, plaque-type psoriasis, psoriatic arthritis, and inflammatory bowel disease [18-20]. Significant cost savings were reported with non-medical switching to biosimilars in a variety of clinical conditions [2].

## Conclusions

This is the first case report in the medical literature that shows non-medical switching can cause DKA. Non-medical switching can cause confusion about the treatment regimen. We believe the patient’s medical team, and not the insurance company or the pharmacy benefit manager, should recommend treatment regimens. We show that non-medical switching does not always save time and can increase spending associated with a preventable hospital admission. To reach realistic conclusions about the cost-effectiveness of non-medical switching, future investigations should focus on comparing the total cost of healthcare spending instead of focusing only on drug prices.
